# Supplementary material for: A chromosome-level genome assembly of longnose gar, Lepisosteus osseus
Source: G3 (Bethesda). 2023 Apr 29;13(7):jkad095. doi: 10.1093/g3journal/jkad095 (PMC10320754; doi:10.1093/g3journal/jkad095)
Supplement: jkad095_Supplementary_Data [file jkad095_supplementary_data.pdf]

Supplemental Tables and Figures

Genome Biology and Evolution

A chromosome level genome assembly of longnose gar, *Lepisosteus osseus*

Rittika Mallik[1*], Kara B. Carlson[2], Dustin J. Wcisel[2], Michael Fisk[3], Jeffrey A. Yoder[2], Alex Dornburg[1]

1. *Department of Bioinformatics and Genomics, UNC-Charlotte, Charlotte, NC USA*
2. *Department of Molecular Biomedical Sciences, Genetics and Genomics Academy, and Comparative Medicine Institute, North Carolina State University, Raleigh, NC, USA*
3. *Aquatic Wildlife Diversity Group, North Carolina Wildlife Resources Commission, Raleigh, NC*

* Corresponding author:
Rittika Mallik
rmallik1@uncc.edu

Contents:

Supplemental Table S1. Genome assembly statistics of longnose gar. 2

Supplemental Table S2. BUSCO analysis of the longnose gar genome assembly. 2

Supplemental Table S3. BUSCO analysis of the longnose gar transcriptome assembly. 3

Supplemental Figure S1. Summary of Gene Ontology Molecular Functions analysis from the longnose gar transcriptome. 4

Supplemental Figure S2. Summary of Gene Ontology Biological Processes analysis from the longnose gar transcriptome. 5

Supplemental Figure S3. Summary of Gene Ontology Cellular Components analysis from the longnose gar transcriptome. 6

Supplemental Table S1. Genome assembly statistics of longnose gar

Feature	Value
GC content	40.1%
Number of Scaffolds	22,745
Number of scaffolds >1 kbp	22,709
Contig N_{50}	116.61 kb
Scaffold N_{50}	52.996Mb
Scaffold L_{50}	8
L_{90}	26 scaffolds
N_{90}	5.560 Mb
Longest scaffold	74,198,471 bp
Number of gaps	27,358
Percent of genome in gaps	2.45%

Supplemental Table S2. BUSCO analysis of the longnose gar genome assembly

Feature	Actinopterygii	Vertebrata
Complete BUSCOs (C)	3017 (82.8%)	2898 (86.4%)
Complete and single-copy BUSCOs (S)	2957 (81.2%)	2867 (85.5%)
Complete and duplicated BUSCOs (D)	60 (1.6%)	30 (0.9%)
Fragmented BUSCOs (F)	65 (1.8%)	97 (2.9%)
Missing BUSCOs (M)	558 (15.4%)	359 (10.7%)
Total BUSCO groups searched (n)	3640	3354

Supplemental Table S3. BUSCO analysis of the longnose gar transcriptome assembly

Feature	Actinopterygii	Vertebrata
Complete BUSCOs (C)	2809 (77.2%)	2792 (83.3%)
Complete and single-copy BUSCOs (S)	1524 (41.9%)	1492 (44.7%)
Complete and duplicated BUSCOs (D)	1285 (35.3%)	1294 (38.6%)
Fragmented BUSCOs (F)	187 (5.1%)	214 (6.4%)
Missing BUSCOs (M)	644 (17.7%)	348 (10.3%)
Total BUSCO groups searched (n)	3640	3354

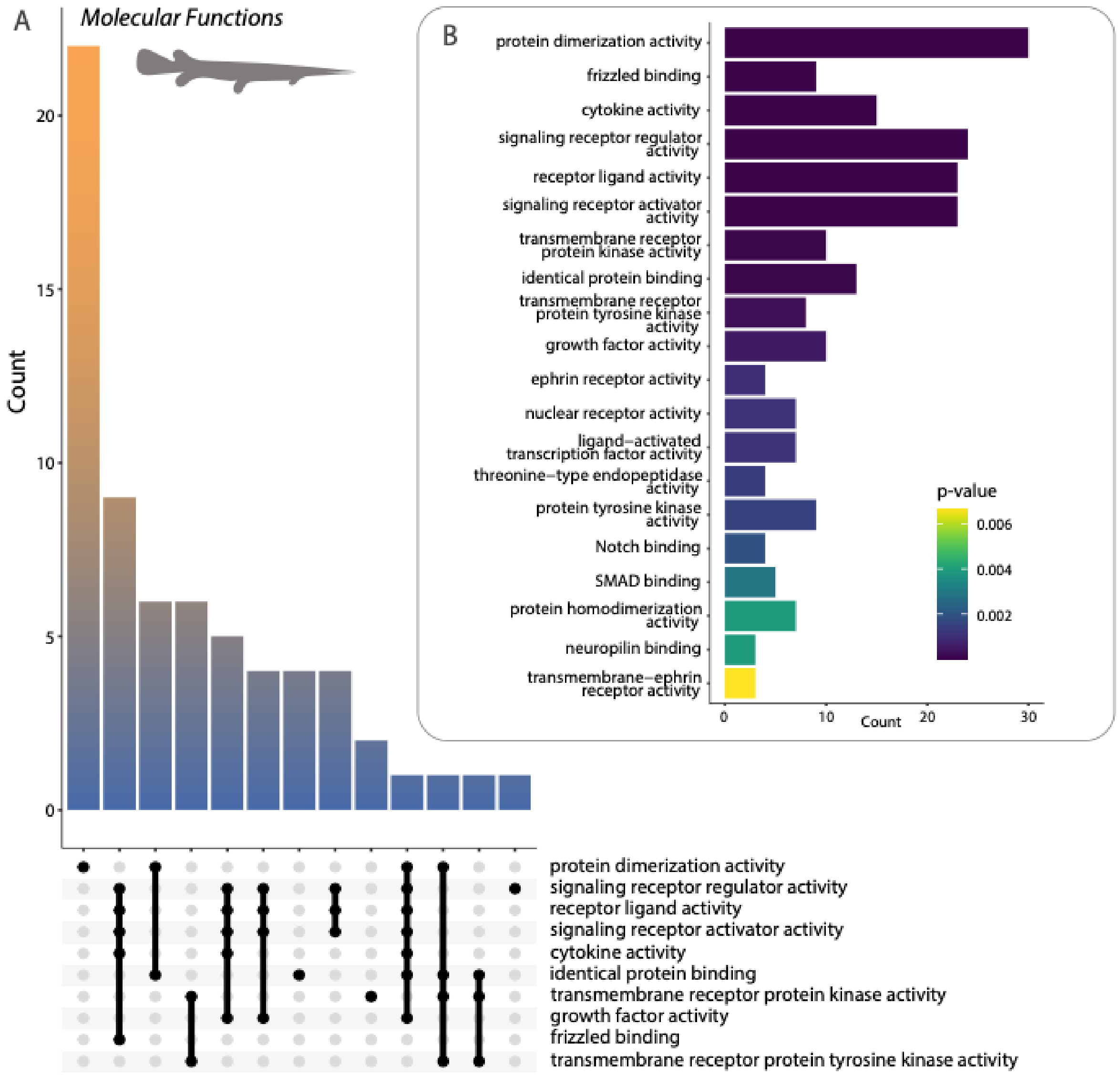

Supplemental Figure S1. Summary of Gene Ontology *Molecular Functions* analysis from the longnose gar transcriptome.
Predicted proteins from the transcriptome were used as inputs to assess (A) functions and their intersections and (B) most common terms.

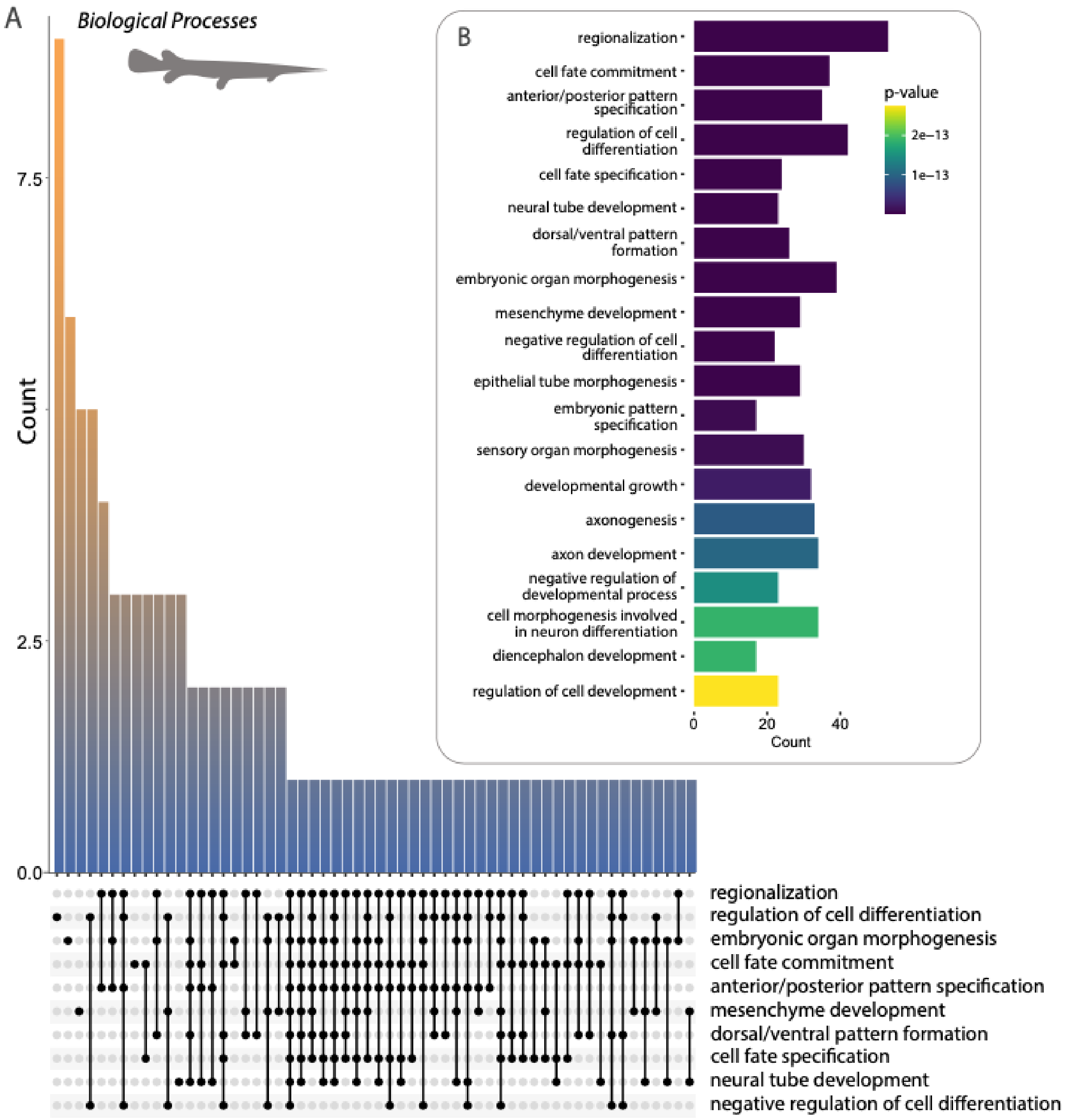

Supplemental Figure S2. Summary of Gene Ontology *Biological Processes* analysis from the longnose gar transcriptome.
Predicted proteins from the transcriptome were used as inputs to assess (A) processes and their intersections and (B) most common terms.

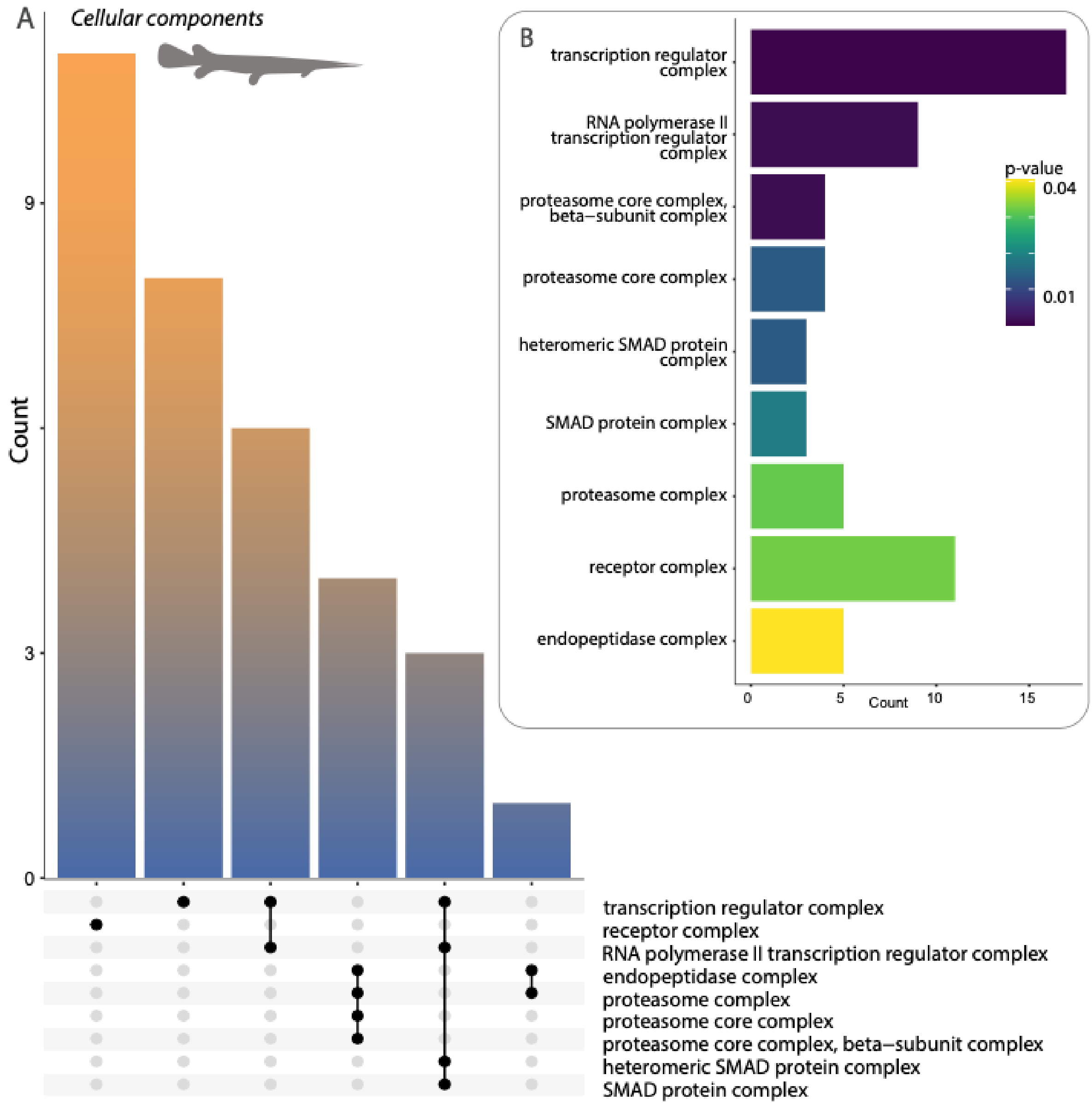

Supplemental Figure S3. Summary of Gene Ontology *Cellular Components* analysis from the longnose gar transcriptome.
Predicted proteins from the transcriptome were used as inputs to assess (A) components and their intersections and (B) most common terms.
